# Possible immune mechanisms of gut microbiota and its metabolites in the occurrence and development of immune thrombocytopenia

**DOI:** 10.3389/fmicb.2024.1426911

**Published:** 2024-08-07

**Authors:** Gengda Zhu, Lixiang Yan, Lijun Fang, Chenyang Fan, Hui Sun, Xinli Zhou, Yucheng Zhang, Zhexin Shi

**Affiliations:** ^1^National Medical Research Center of Acupuncture and Moxibustion, First Teaching Hospital of Tianjin University of Traditional Chinese Medicine, Tianjin, China; ^2^Institute of Hematology and Blood Diseases Hospital, National Clinical Medical Research Center for Blood Diseases, Tianjin, China

**Keywords:** gut microbiota, metabolites, pathogenesis, immune thrombocytopenia, intestinal barrier, leaky gut

## Abstract

Immune thrombocytopenia (ITP) is an autoimmune disease characterized by increased platelet destruction and impaired production, leading to an elevated bleeding tendency. Recent studies have demonstrated an important link between the gut microbiota and the onset and progression of several immune diseases in humans, emphasizing that gut microbiota-derived metabolites play a non-negligible role in autoimmune diseases. The gut microbiota and its metabolites, such as short-chain fatty acids, oxidized trimethylamine, tryptophan metabolites, secondary bile acids and lipopolysaccharides, can alter intestinal barrier permeability by modulating immune cell differentiation and cytokine secretion, which in turn affects the systemic immune function of the host. It is therefore reasonable to hypothesize that ecological dysregulation of the gut microbiota may be an entirely new factor in the triggering of ITP. This article reviews the potential immune-related mechanisms of the gut microbiota and representative metabolites in ITP, as well as the important influence of leaky gut on the development of ITP, with a view to enriching the theoretical system of ITP-related gut microecology and providing new ideas for the study of ITP.

## 1 Introduction

Immune thrombocytopenia (ITP) is an autoimmune disease mainly mediated by platelet autoantibodies, characterized by multiple hemorrhages and scattered petechial ecchymoses on the skin and mucous membranes, as well as reduced platelet counts (Lambert and Gernsheimer, 2017). The pathogenesis of ITP is complex, with increased platelet destruction mediated by humoral immunity being the cause of most cases. In patients with ITP, plasma cells produce a large number of IgG-based autoantibodies, which bind to the Fcγ receptor on the surface of macrophages in the spleen and mediate the destruction of platelets labeled with anti-glycoprotein autoantibodies, leading to the formation of ITP (Cooper and Ghanima, 2019). In some patients with ITP, no autoantibodies to platelet membrane glycoproteins have been detected, and abnormalities in their cellular immune mechanisms are also an important part of the pathogenesis of ITP, including impaired megakaryopoiesis, Th1/Th2 imbalance, alteration of the ratio of helper T-cells, macrophage and dendritic cell messaging, CD8+ T-cells inducing platelet destruction either directly or indirectly, and a dysfunction of the balance of Th17/Tregs, etc. (Swinkels et al., 2018). In recent years, the study of various autoimmune diseases and gut microbiota has become a hot spot, and with the continuous development and application of multi-omics technology, high-throughput sequencing technology and bioinformatics technology, human interest in the composition and function of the gut microbiota has become increasingly widespread and closely. Numerous studies have shown that the gut microbiota has a profound impact on the development, growth and function of multiple host systems. The gut microbiota is closely associated with diseases such as type 1 diabetes, rheumatoid arthritis, systemic lupus erythematosus and autoimmune hepatitis in terms of immunity by regulating immune cell differentiation and cytokine secretion (Chu et al., 2021; Zhang et al., 2021; Del Chierico et al., 2022; Sun et al., 2024). Recently, a growing body of research has identified the gut microbiota as a possible class of factors that may influence the development of ITP. However, the role of gut microbiota and its metabolites in ITP is still unclear, and most of the research focus still remains in basic experiments, and the gut microbiota is far away from being used in the treatment of ITP. In this review, we first summarize the characteristics of the gut microbiota in patients with ITP. In addition, potential specific mechanisms by which the gut microbiota and its metabolites are involved in regulating the immune environment in ITP patients are discussed.

## 2 Dysregulation of gut microbiota in patients with immune thrombocytopenia

The organ with the largest distribution of microorganisms in the human body is the intestine, and the composition of the intestinal microbiota is complex and diverse, including bacteria, fungi, viruses, and protozoa, among which bacteria dominate, with a mass of about 0.2 kg (Sender et al., 2016; Yang and Cong, 2021). The intestinal microbiota is mainly composed of six phyla, including Firmicutes, Bacteroidetes, Actinobacteria, Proteobacteria, Clostridium, and Verrucomycota, with Firmicutes and Bacteroidetes as the main types (Hou et al., 2022). The normal human intestinal microbiota has a role in regulating the metabolism of human substances and participating in the regulation of the host immune response, as well as being an important part of the developmental process of the intestinal mucosal immune system (Sebastián Domingo, 2018). For example, *Clostridium difficile* induces Treg cells, and Segmented Filamentous Bacteria (SFB) and Citrobacter induces the development of functionally distinct Th17 cell populations in the gut, respectively (Ivanov et al., 2009; Omenetti et al., 2019).

There is growing evidence that the gut microbiota of ITP patients is specific. Liu et al. (2020) showed an increase in the proportion of Ascomycetes and a decrease in the proportions of Firmicutes (F), Actinobacteria (A), and Firmicutes/Bacteroides (F/B) in ITP patients; the same results were also demonstrated by Yu X. et al. (2022) in a study by Liu et al. (2020). However, in Zhang et al.’s (2020) study, the proportions of Actinobacteria, Streptococcus, and Lactobacillus were increased in ITP patients, while Bacteroides was massively decreased, which is in complete contrast to previously reported results. Wang et al. (2021) found that at the phylum level, Firmicutes and Bacteroidetes in ITP patients were not significantly different from normal subjects, but differences in genus and species still existed. In particular, the genus and species of the gut microbiota of corticosteroid-resistant ITP patients show a richer heterogeneity compared to corticosteroid-sensitive ITP patients (Wang et al., 2021). A small study of pediatric ITP found that the gut microbiota of ITP patients had higher proportions of Firmicutes and Bacteroidetes phage and lower proportions of Actinobacteria and Proteobacteria phage compared to healthy volunteers, and a positive correlation was found between Bacteroidetes phage and IgG, suggesting that the gut microbiota may be involved in the pathogenesis of ITP by influencing IgG. Using Mendelian randomization to analyze the data, Guo et al. (2023) showed that the proportion of associated flora belonging to the phylum Firmicutes and the genus Actinobacteria decreased in ITP patients, but the causal relationship between ITP and the gut microbiota was still unclear (Li et al., 2023). Guo et al. (2023) showed a decrease in Firmicutes, an increase in Proteobacteria, and a difference in multiple genera and species in patients with ITP. And plasma IL-2 concentrations were higher in ITP patients than in healthy individuals, but there was no significant difference in IL-4 (Wang H. et al., 2023). Although all of the above researchers have paid attention to the changes of gut microbial species in ITP patients, their results are not yet consistent, which may be due to factors such as grading and staging of the disease, differences in age, diet, environment, gender, and methodological bias of the study participants. At present, the specific mechanism of gut microbiota in the occurrence and development of ITP is still unclear, and the above studies mainly focused on the direct effect of gut microbiota on ITP, neglecting the potential value of gut microbiota metabolites. Therefore, it is essential to clarify the relationship between gut microbiota metabolites and ITP (Table 1).

**Table 1:** Changes in the gut microbiota in patients with immune thrombocytopenia.

Researches	Participant	Gut microbiota in patients with ITP	The role of the gut microbiota	Reference
Wang et al.	ITP (99) HC (52)	(treatment-naïve ITP patients) Phyla: Actinobacteria↑, Fusobacteria↑, Verrucomicrobia↑. Species: *Bacteroides coprophilus*↑, *Ruminococcus gnavus*↑, *Bifidobacterium longum*↑, *Akkermansia muciniphila*↑. (corticosteroid-resistant ITP patients) Genus: Pedobacter↑, Lachnobacterium↓, and Rhodonellum↓, Species: *Bifidobacterium scardovii*↑, *Clostridium tyrobutyricum*↑.	Compared to the healthy population, the gut microbial community structure of treatment-naïve ITP patients did not differ significantly at the portal level, but varied more at the genus and species levels. Gut microbiota function differently in corticosteroid-resistant and treatment-naïve patients.	Wang et al. (2021)
Liu et al.	ITP (94) HC (93)	Phyla: Proteobacteria↑, Firmicutes↓, Actinobacteria↓, Firmicutes/Bacteroidetes ratio↓.	Significant activation of platelet markers PAC-1 and CD62p.	Liu et al. (2020)
Yu et al.	ITP (29) HC (33)	Phyla: Proteobacteria↑, Chloroflexi↑, Firmicutes↓, Actinobacteria↓, Firmicutes/Bacteroidetes ratio↓.	IL-6↑, TNF-α↑.	Yu X. et al. (2022)
Zhang et al.	ITP (30) HC (29)	Phyla: Bacteroidetes↓, Firmicutes/Bacteroidetes ratio↑. Species: Actinobacteria↑, Streptococcus↑, Lactobacillus↑.	*S. anginosus*, Cer (t18:0/16:0), Cer (d18:1/17:0) and 13-hydroxyoctade canoic acid showed a highly negative correlation with platelet counts.	Zhang et al. (2020)
Li et al.	ITP (25) HC (16)	Phyla: Firmicutes/Bacteroidetes ratio↑, Actinobacteria/Proteobacteria ratio↓.	Positive correlation between Bacteroidetes and IgG.	Li et al. (2023)
Guo et al.	ITP (337) HC (408)	Phyla: Firmicutes↓, Actinobacteria↓.	Alcaligenaceae and Methanobacteriaceae were causally associated with ITP, while ITP did not significantly affect the gut microbiota.	Guo et al. (2023)
Wang et al.	ITP (40) HC (33)	Phyla: Bacteroidetes↓, Proteobacteria↑. Genus: 55 genera differ in relative abundance.	Plasma IL-2 concentrations were higher in ITP patients than in HC; There was no significant difference in plasma IL-4 between the two groups.	Wang H. et al. (2023)

HC, healthy control; ITP, immune thrombocytopenia; Cer, ceramide; IL, interleukin; CD62p, P-selectin; TNF, tumor necrosis factor.

## 3 Potential link between gut microbiota metabolites and ITP

Alterations in the gut microbiota can participate in the generation of the host immune system and in a range of inflammatory responses, and the metabolites produced by the gut microbiota, as the signaling molecules, can be seen as mediators of the interactions between the gut microbiota and their hosts. Gut microbiota metabolites can be regarded as mediators of the interaction between gut microbiota and their hosts, and some gut microbiota metabolites have antioxidant, anti-inflammatory, and immune cell-regulating activities, which may be potentially beneficial for the treatment of ITP (Liu et al., 2020; Liu X. G. et al., 2023). However, studies on the metabolites of the gut microbiota in ITP patients are lacking and require further research and refinement, and the regulation of immune cell differentiation and cytokine secretion by gut microbiota metabolites is comprehensively described below.

### 3.1 Short-chain fatty acids

Short-chain fatty acids (SCFAs) are mainly produced by intestinal anaerobes fermenting dietary fiber and absorbed carbohydrates, including acetic acid, propionic acid, and butyric acid, and are inhibitors of histone deacetylase (HDAC) and G-protein-coupled receptors (GPCRs). GPCRs ligands, with butyric acid being the most widely studied. Bacteria of the phylum Anabaena mainly produce large amounts of acetate and propionate, whereas bacteria of the phylum Thickettsia are mainly responsible for butyrate production (Blaak et al., 2020). Several studies have shown that butyrate can inhibit the secretion of inflammatory factors such as IL-12 and TNF-α through several signaling pathways such as NF-κB, and most studies have concluded that the proportion of thick-walled bacterial phylum in the intestines of patients with ITP is on the decline, which is consistent with the pathogenesis of ITP. Gut microorganisms are involved in mediating many physiological processes between hosts through short-chain fatty acids, including maintenance of gut barrier function, immunomodulation, and anti-inflammatory effects. Lipids and lipid-like molecules are closely associated with different gut microbiota and may be involved in the pathogenesis of ITP (Macfarlane and Macfarlane, 2003; Zhang et al., 2020; Yang and Cong, 2021; Liu X. F. et al., 2023).

#### 3.1.1 Involved in the development, differentiation and functional expression of various T cells

Furusawa et al. (2013) found that their intestinal microbiota was able to upregulate the expression of Tregs in mouse experiments. This study found that the bacterial metabolite butyrate regulates the expression of genes related to T cell differentiation and promotes the differentiation and maturation of Tregs by inhibiting HDAC expression through GPR43 (Furusawa et al., 2013). Chen et al. (2019) found that butyrate promotes Th1 cell development by upregulating the expression of the transcription factor T-bet and inhibits Th17 cell differentiation by suppressing Rorγt and other Th17-related transcription factors. In addition, butyrate contributes to the maintenance of intestinal homeostasis by regulating the development and differentiation of Th1 and Th17 cells while inducing the production of IL-10 by both to achieve the ability to inhibit T cells from inducing colitis (Chen et al., 2019). Whereas a decrease in the proportion of thick-walled bacterial phylum leads to a decrease in butyrate production, this pro-inflammatory tendency may be linked to the pathogenesis of ITP development. In a study by Zhao et al. (2019) ITP may be associated with aberrant histone acetylation of the CTLA4 gene H3K27, which was less acetylated in ITP patients than in controls, suggesting that the reduction of SCFAs in the gut of ITP patients inhibits the differentiation of Tregs. It has been shown that SCFAs can also participate in the differentiation of CD4+ T cells, promoting their differentiation into Th1 cells by inhibiting HDAC activity and activating the mTOR-S6K pathway (Furusawa et al., 2013). In addition, SCFAs can promote IL-22 production by type 3 innate lymphocytes (ILC3) and some CD4+ T cells through the expression of aromatic hydrocarbon receptors and hypoxia-inducible factor 1α (Yang et al., 2020). This implies that butyrate may modulate various genes to exert its full range of anti-inflammatory effects.

#### 3.1.2 Regulation of B-cell-associated antibody secretion

The SCFAs have been shown to be involved in the process of B-cell activation and antibody production. SCFAs directly increase the level of acetyl coenzyme a in B-cells, promoting fatty acid synthesis, glycolysis and energy production for the tricarboxylic acid cycle, which favors the secretion of antibodies (Kim et al., 2016). It has been shown that after feeding different doses of prebiotics to experimental mice, the levels of SCFAs and IgA in the intestines increased with the dose, as well as the levels of IgA and IgG in the serum of the mice, which suggests that SCFAs can affect the antibody levels throughout the host’s body. In addition, SCFAs alter B-cell-related genes that are involved in B-cell differentiation and influence antibody production, including IgG and IgA (Kim et al., 2016; Liu X. F. et al., 2023).

#### 3.1.3 Regulation of dendritic cells and macrophages affects cytokine and chemokine production

The SCFAs can affect cytokine or chemokine production by regulating dendritic cells. In germ-free mice, not only are macrophages and dendritic cells unable to produce interleukins such as TNF, IFN, and IL-10, but they also indirectly inhibit T-cell activation and maturation, suggesting that SCFAs are an essential and important component in the development of the host immune system (Jiao et al., 2020). Macrophage polarization is one of the key hubs of the local inflammatory response, and it has been shown that SCFAs selectively activate the polarization of macrophages to enhance the body’s anti-inflammatory function (Lu et al., 2022). In addition, an increase in the number of macrophages and dendritic cells was observed in mice treated with propionate, which altered bone marrow hematopoiesis (Trompette et al., 2014; Jiao et al., 2020). This implies that SCFAs have the potential to be used as therapeutic agents for hematologic diseases such as ITP.

### 3.2 Trimethylamine *N*-oxide

Trimethylamine (TMA) is an intestinal microbial metabolite derived mostly from dietary carnitine and choline. TMA is absorbed from the intestines and transferred to the liver where it is oxidized to trimethylamine-*N*, which is oxidized by flavin monooxygenase enzymes (FMOs) to form trimethylamine *N*-oxide (TMAO). TMAO has been demonstrated to be a potential pathogenetic factor in rheumatoid arthritis and systemic lupus erythematosus TMAO induces polarization of M1 macrophages and can directly modulate platelet hyperreactivity and clot formation rates *in vivo* to influence platelet activation (Wang J. et al., 2023; Liu et al., 2024). Liu et al. (2024) found that peripheral plasma concentrations of TMAO were significantly reduced in ITP patients, a trend that is different from some autoimmune diseases and not observed in other autoimmune diseases. It was also observed that corticosteroid-resistant patients exhibited a significant TMAO profile compared to sensitized individuals, suggesting a possible role of TMAO in corticosteroid resistance in ITP. Whereas IL-1β and TNF-α were significantly increased in ITP patients, suggesting that TMAO may mediate corticosteroid resistance in ITP patients through oxidative stress. And Wang et al. (2021) showed that seven out of nine depleted gut microbiota identified in corticosteroid-resistant patients belonged to the phylum Thickettsia (Skye et al., 2018). Therefore, it is reasonable to hypothesize that there is a close association between the reduced proportion of Firmicutes, elevated levels of TMAO and corticosteroid resistance in ITP patients.

### 3.3 Tryptophan metabolites

Tryptophan (Trp) is one of the essential amino acids, which can be metabolized into different metabolites by intestinal microbiota, intestinal epithelial cells and immune cells. Gut microbes can produce tryptophan metabolites like indole acetic acid (IAA) by metabolizing Trp or kynurenine (Kyn) pathways (Su et al., 2022; Wang J. et al., 2023). Tryptophan metabolites were found to maintain immune homeostasis in the gut and host organism by promoting the functional expression of immune cells such as T cells, B cells, phagocytes and ILC3, a finding that has important implications for the treatment of ITP and other autoimmune diseases (Su et al., 2022; Wang J. et al., 2023). It was found that IAA could reduce Th17 cell production by activating the aromatic hydrocarbon receptor (AhR) and down-regulating RORγt and STAT3 (Su et al., 2022), suggesting that IAA has the potential to be a targeted drug for the treatment of ITP. However, according to Choi et al. (2020), lupus-susceptible mice had higher serum and fecal levels of kynurenine than controls, and the immune-inflammatory response was attenuated on a low-tryptophan diet, suggesting that tryptophan metabolites may induce the activation of autoimmunity, which is contrary to the function of tryptophan metabolites produced by the gut microbiota. Therefore further exploration and validation in ITP animal models or even clinical trials are needed.

### 3.4 Secondary bile acids

Secondary bile acids (SBAs) are a class of products formed by the metabolism of the gut microbiota when primary bile acids flow through the gut, including deoxycholic acid and lithocholic acid. Bile acids are agonists of farnesoid X receptor (FXR) and play an important role in regulating multiple antimicrobial pathways in the gut (Guzior and Quinn, 2021). Studies have shown that secondary bile acids induce Treg differentiation and RORγ+ Treg cell development, but inhibit Th17 cell differentiation (Jia et al., 2023). Hang et al. (2019) found that two different derivatives of lithocholic acid (LCA), 3-oxolithocholic acid (3-oxoLCA) and isocholic acid (isoalloLCA), modulate the differentiation of Th17 and Treg cells.3-oxoLCA inhibits Th17 cell differentiation by binding to the transcription factor RORγt, whereas isoalloLCA promotes Treg differentiation by generating mitochondrial reactive oxygen species and leads to increased expression of FoxP3 (Hang et al., 2019). FoxP3 serves as an important transcription factor for Treg, which implies that secondary bile acids, which are metabolized by the intestinal microbiota, may, by affecting Treg expression mediating the development of ITP. Paik et al. (2022) also further demonstrated that gut microbiota metabolizes lithocholic acid to 3-oxoLCA and isoalloLCA, both of which function to inhibit Th17 cell expression. In addition, Campbell et al. (2020) found that the secondary bile acid 3β-hydroxydeoxycholic acid enhances Foxp3 expression by acting on dendritic cells to reduce their immunostimulatory properties in order to promote the production of Tregs (Campbell et al., 2020).

### 3.5 Lipopolysaccharide

Lipopolysaccharide (LPS), also known as endotoxin, consists of lipids and polysaccharides, a macromolecule found predominantly in the outer wall of the cell wall of Gram-negative bacteria, which activates the innate immune response by binding to Toll-like receptor 4 (TLR4). In addition to dietary intake, a large number and variety of intestinal bacteria may be a major source of blood LPS. In healthy individuals, the intestinal barrier prevents LPS from entering the circulation. When the intestinal microbiota is dysbiotic, the elevated permeability of the intestinal barrier allows LPS to enter the bloodstream, thereby inducing inflammation and immune responses. Polysaccharide is also considered as an immunomodulator, which activates the immune system and participates in immune responses such as antimicrobial, antiviral and antitumor (Tanaka et al., 2022). It has been suggested that when LPS binds to TLR4, it stimulates the expression of TNF-α, IL-12, and IFN-β through the bridging protein-myeloid differentiation factor 88 and NFκB signaling pathways, thereby stimulating the differentiation of T cells to Th1 and Th17.LPS also stimulates the release of IL-6 and TNF-α from B cells, as well as increasing their antibody production (Zhou et al., 2014). This study concluded that LPS induces IL-8 expression and directly impairs the intestinal epithelial barrier, which may be one of the co-factors in the formation of autoimmune diseases (Campbell et al., 2020). However, Brown et al. (2023) showed that LPS isolated from Bacteroidetes spp. does not overly produce an inflammatory response, due to alterations in its lipid A acyl chains that result in different mediated TLR2 and TLR4 signaling. Vatanen et al. (2016) similarly found that LPS produced by Bacteroidetes spp. is structurally different from LPS produced by *Escherichia coli*, with the former having the ability to inhibit innate immune signaling and activate endotoxin tolerance. Transmission and activation of endotoxin tolerance. In addition, this study observed that the sources of lipopolysaccharide produced by the gut microbiota were not the same in different geographical populations (Candelli et al., 2021). It was also found that LPS can produce inflammatory factors, such as IL-6 and IL-12, by stimulating dendritic cells and macrophages, which may trigger the development of effector T-cells and T-cell-mediated immune responses (Pamuk et al., 2023). Thus, it is evident that lipopolysaccharides produced by the gut microbiota play an important role in participating in the immune process, which may influence the development of various autoimmune diseases such as ITP in addition to IBD, and that the mechanism of action of LPS from different sources varies.

## 4 Potential mechanism of influence of gut microbiota flora and its metabolites on the pathogenesis of immune thrombocytopenia

**Figure 1:**
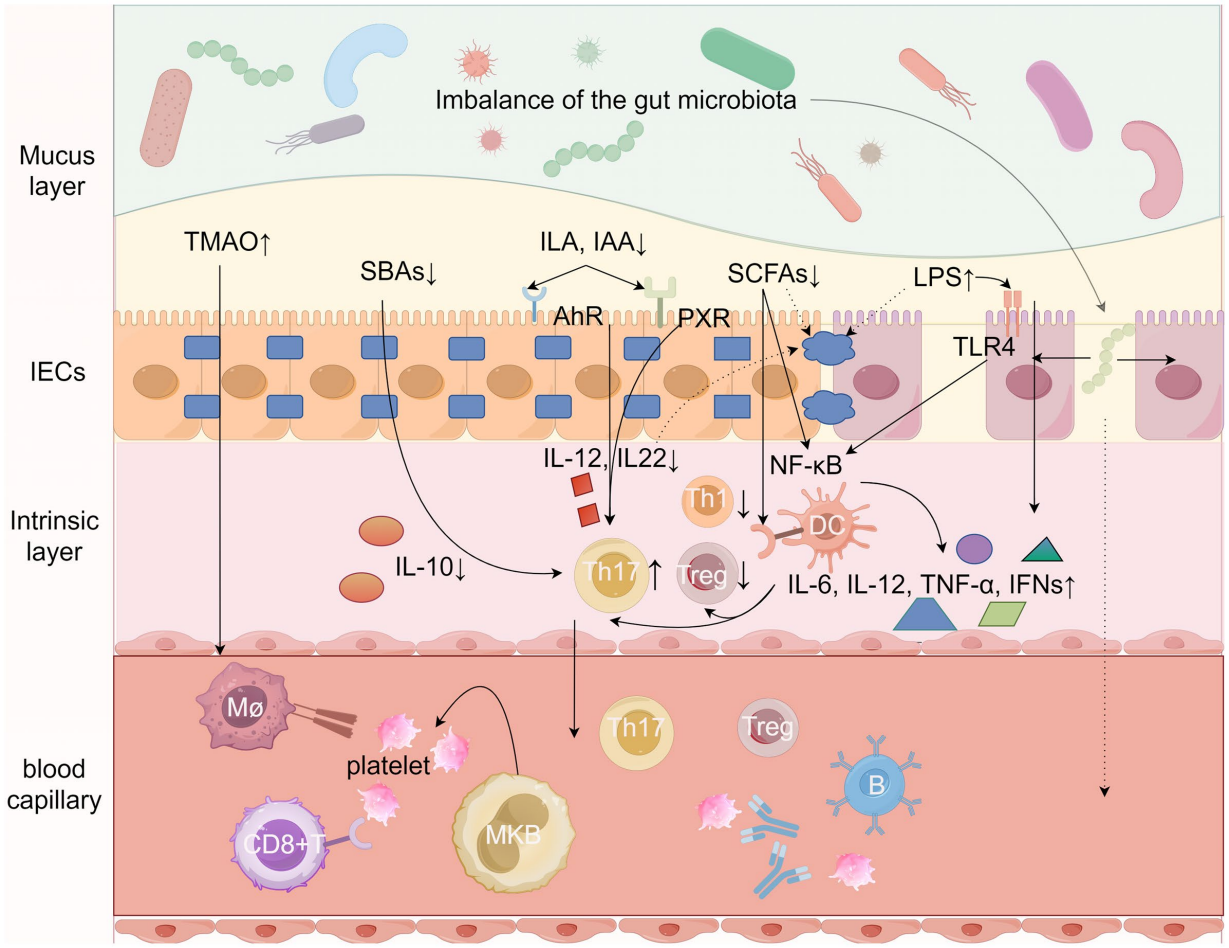
Potential mechanisms by which gut microbes and their metabolites influence ITP. Gut microbes and their metabolites such as short-chain fatty acids (SCFAs), trimethylamine N-oxide (TMAO), secondary bile acids (SBAs), Tryptophan metabolites, and LPS from Gram-negative bacteria collectively stimulate both the intrinsic immune system (dendritic cells, macrophages, and neutrophils) and the adaptive immune system (B cells and T cells) by Regulate the secretion of a variety of cytokines, alter intestinal barrier permeability, and enter the circulatory system to directly or indirectly mediate the onset and development of ITP. LPS, lipopolysaccharides; SCFAs, short-chain fatty acids; TMAO, trimethylamine N-oxide; SBAs, secondary bile acids; ILA, indole-3-lactic acid; IAA, indoleacetic acid; AhR, aromatic hydrocarbon receptor; Th1, helper T-cell 1; Th17, helper T-cell 17; Treg, regulatory T-cell, TLR4, toll-like receptor 4; NF-kB, nuclear factor-κB; mø, macrophage, IL, Interleukin; PXR, pregnane X receptor. Figure by Figdraw.

The pathology of ITP has been studied specifically for more than one hundred years, and then existing studies still cannot fully explain the specific pathogenesis of ITP. Numerous studies today have found or demonstrated that the gut microbiota is inextricably linked to the development of autoimmune diseases (Jiang et al., 2022; Zou et al., 2022), based on the current available knowledge we can propose a new model that illustrates the potential specific mechanisms by which the gut microbiota and its metabolites influence the pathogenesis and development of ITP, such as alteration of gut microbiota metabolites, imbalance of gut immune homeostasis, increased gut barrier permeability, gut microbiota translocation, etc. (Paray et al., 2020; Pan et al., 2021; Zhang et al., 2021; Peh et al., 2022; Aleman et al., 2023; Campbell et al., 2023; Miyauchi et al., 2023; Stec et al., 2023; Yao et al., 2023) (Figure 1).

### 4.1 Impaired intestinal barrier and leaky gut

The exact pathogenesis of ITP remains unclear, but increasing evidence suggests that gut barrier damage and leaky gut production may be an important factor (Kinashi and Hase, 2021; Usuda et al., 2021; Christovich and Luo, 2022). The intestinal barrier, which consists of four barriers: microbial, chemical, mechanical and immune, plays an important protective role in isolating bacteria and harmful substances (Di Tommaso et al., 2021; Yu S. et al., 2022). Intestinal epithelial cells (IECs) are the main body of the intestinal barrier and act as a physical barrier. The connections between IECs are sealed by tight junctions (TJs).The intestinal epithelium is topped by a mucus layer. The large intestine is divided into an inner and an outer layer, which is dominated by mucin. The outer layer contains a variety of microorganisms, the inner layer is virtually free of microorganisms, and there is only a single layer in the small intestine in which the PAN cells located in the small intestine secrete antimicrobial peptides, defensins and other immunomodulatory proteins that limit the attack of bacteria and harmful substances on the intestinal epithelial cells (Martel et al., 2022). A variety of protein molecules control tight junctions, which are formed abnormally or loosely under various pathological conditions, leading to the entry of a variety of potentially harmful molecules into the organism. The lower part of the intestinal epithelium is the lamina propria, which includes Pyle’s nodes and isolated lymphoid follicles, and immune cells, such as B cells, T cells, dendritic cells (DCs), neutrophils, and macrophages, which regulate immunity by presenting antigens, secreting cytokines, and producing relevant antibodies, and together with a large amount of secreted IgA, form the immune barrier. The integrity of the intestinal barrier is closely related to intestinal epithelial cells, intercellular tight junctions, and the intestinal microbiota (Mu et al., 2017; Camilleri, 2019). Leaky gut has not yet been accurately defined, and can be understood as a state of intestinal ecological dysregulation in which microorganisms and their metabolites in the gut enter the lamina propria and the bloodstream unrestrictedly after the intestinal barrier has been damaged to a certain degree, and a disturbed gut microbiota can cause damage to the intestinal barrier (Chelakkot et al., 2018). After leaky gut occurs, intestinal microbes and their metabolites can affect platelet production and destruction by stimulating intestinal immune cells, generating relevant effector cells and many signaling factors, and subsequently diffusing throughout the body via capillaries. Of course, some intestinal microorganisms and their metabolites can enter the bloodstream and directly stimulate peripheral lymphoid organs to produce autoreactive immune cells.

### 4.2 Influence of gut microbial metabolites on the intestinal barrier

The integrity of the intestinal barrier function is closely related to the LPS of Gram-negative bacteria, metabolites of the dominant flora, such as SCFAs and Trp metabolites, and intestinal inflammatory factors, including TNF-α, INF-γ, IL10, and so on (Di Tommaso et al., 2021; Martel et al., 2022). The intestinal microbiota is important for the formation of the intestinal immune system, and some probiotics not only favor the maturation and differentiation of intestinal epithelial cells and various immune cell subpopulations, but also promote the production of secretory IgA (Usuda et al., 2021). It has been found that the interaction between LPS and TLR exacerbates the damaging effects of the inflammatory response on the intestinal barrier, and the occurrence of leaky gut may lead to the entry of LPS into the circulatory system, which in turn affects the immune function of the host organism (Mu et al., 2017; Camilleri, 2019). Both TNF-α and IFN-γ increase intestinal permeability by impairing tight junctions (Srikantha and Mohajeri, 2019).

The SCFAs are critical for the maintenance of intestinal bacterial homeostasis, intestinal epithelial functional integrity, intestinal immunity and inflammation (Rooks and Garrett, 2016; Yoo et al., 2020). SCFAs can increase mucus layer production by regulating the transcription of mucin genes in the cuprocytes (Abdelhamid and Luo, 2018). It was shown that NOD-like receptor protein 3 (NLRP3) inflammasome-mediated pyroptosis is thought to damage the intestinal barrier, and SCFAs can protect and repair the damaged intestinal barrier by inhibiting NLRP3 inflammasome and autophagy. Butyrate is a frequently investigated SCFA metabolite that, in addition to decreasing intestinal inflammation and injury, additionally affects tight junction proteins by up-regulating the expression of ZO-1, occludin, and tight junction protein-4 in order to maintain intestinal mucosal integrity (Miao et al., 2016; Takeuchi and Okamoto, 2022). In addition, intestinal bacteria or bacterial components can promote autoantibody production through molecular mimicry, like *Helicobacter pylori*. As early as the end of the 20th century, research found that eradicating *Helicobacter pylori* would significantly increase platelet counts (Gasbarrini et al., 1998). In the following 20 years, there have been reports and clinical observations of cases of ITP caused by *Helicobacter pylori* (Franchini et al., 2007; Sadia et al., 2022). Excitingly, these studies all indicate and emphasize the significant increase in platelet count after eradicating *Helicobacter pylori* (Lee et al., 2020). It can simultaneously react with platelet surface antigens to produce cross-reactive antibodies through molecular mimicry, which exacerbates platelet destruction inducing the development of ITP (Morton et al., 2014). *Helicobacter pylori* infection may also affect the onset and development of ITP by inducing autoimmunity through urease activation of B-1 cells, altering the balance of monocyte fc γ receptors, and other pathways (Yamanishi et al., 2006; Asahi et al., 2008). Migration of intestinal T cells has been studied in transgenic mice and the results showed that colonic Th17 cells could migrate and transport to the spleen and lymph nodes (Jacob et al., 2020). In addition, gut-derived Treg cells were detected in the pancreas and pancreatic lymph nodes of butyrate-treated non-obese diabetic (NOD) mice, implying that butyrate may promote the migration of Treg cells to the corresponding sites of inflammation (Lanis et al., 2017). These studies suggest that intestinal immune cells can exert some influence on the homeostasis of systemic immunity by migrating to the circulatory system, which may also be a common factor in the pathogenesis of autoimmune diseases.

It has been found that host-produced Trp metabolites such as kynurenine (Kyn) and microbial-produced Trp metabolites such as indole (IND) and indole-3-propionate (IPA) can regulate intestinal barrier function through IL-10 receptors (Lanis et al., 2017; Ye et al., 2022). Ye et al. (2022) showed that indole, when activated by the AHR, secreted IL-22, also maintained normal epithelial barrier function. In addition, indole can regulate intestinal immune homeostasis by activating the pregnane X receptor (PXR) (Ye et al., 2022). Therefore, tryptophan deficiency also affects the integrity of the intestinal barrier, suggesting that tryptophan metabolites may play an important role in the pathogenesis of ITP.

The metabolites of the gut microbiota bind to various pattern recognition receptors, such as TLRs and NLRs. These metabolites can directly stimulate the effector differentiation of B cells by binding to various TLRs on the surface of B cells, including the release of IL-10 and IL-1 β, which are believed to be involved in the pathogenesis of ITP (Couto and Zipfel, 2016). The imbalance of Th17/Treg and Th1/Th2 ratios is an important factor in the pathogenesis of ITP. The gut microbiota promotes the secretion of various interleukins by mediating different types of Toll like receptors, which collectively drive T cell differentiation and expression. However, the results of this change are often negative (Allegra et al., 2023). In addition, TLRs and NLRs on platelets can recognize pathogens and activate related immune responses. When bacteria or related metabolites enter the bloodstream, they can activate TLRs and NLRs receptors on platelets, thereby promoting the release of inflammatory factors. Platelets themselves can also be activated by various metabolic products such as inflammatory factors and TMAO. By releasing effector molecules such as cytokines and chemokines, they activate various immune cells and promote the occurrence and progression of inflammatory reactions (Tang et al., 2023). Research has found that the role of gut microbiota in peripheral anti infection may also depend on agonists of Toll receptors and NOD like receptors entering the body from the gut. For example, peptidoglycan derived from bacteria can better resist *Streptococcus pneumoniae* and *Staphylococcus aureus* in the peripheral circulation of mice by enhancing the innate immune function of the body as an agonist of NOD1 receptors (Clarke et al., 2010). In addition, studies have found that NOD1 receptors can induce the differentiation of bone marrow mesenchymal stem cells into hematopoietic stem cells in sterile mouse experiments (Iwamura et al., 2017). This indicates that pattern recognition receptors not only protect the body from infection and disease invasion, but also have great potential in regulating inflammatory responses, participating in autoimmune diseases, and promoting bone marrow hematopoiesis. They are expected to provide new ideas for drug development and disease treatment.

## 5 Conclusion and expectations

Gut microbiota can be involved in the occurrence and development of many autoimmune diseases through its metabolites, but the study of gut microbiota metabolites in ITP is still in its early stage. By analyzing the association between the gut microbiota and its metabolites and the development of ITP, as well as the monitoring value and prognostic assessment of ITP therapeutic response, the causal relationship between the gut microbiota and its metabolites and the development of ITP can be demonstrated, which will help to provide new targets and pathways for the clinical treatment of ITP. It should be noted that the current research on gut microbiota and its metabolites in autoimmune diseases mainly focuses on basic research and animal experiments, and more clinical studies are needed to validate and clarify the roles in disease progression and treatment, so as to further understand the mechanism of the gut microbiota metabolites in ITP and to evaluate their potential clinical applications, which is particularly important for subsequent research.

At present, there are still many problems in the research related to gut microbiota in ITP, such as whether the type of gut microbiota in ITP patients is statistically different from that of normal people, whether the different degree of ITP disease affects the changes of gut microbiota, whether the different ages, diets, environments, and genders of the study subjects have an effect on the gut microbiota, whether the gut microbiota and its metabolites have an effect on ITP, whether the application value of gut microbiota and its metabolites in ITP is still unclear, and whether the potential clinical application value is still unclear. The application value of gut microbiota and its metabolites in ITP is still unclear, the study on the correlation between gut microbiota metabolites and ITP is still limited, and whether the changes of gut microbiota in different model animals can be representative of human beings and so on. It is believed that with the deepening of the research, the value of the gut microbiota and its metabolites in ITP will be recognized one by one, and its connection with the pathogenesis of ITP and the mechanism of corticosteroid resistance will be further proved. The purpose of studying the immune mechanisms between ITP and gut microbiota and its metabolites is to provide new directions for future diagnostic and therapeutic measures for ITP.

## Author contributions

GZ: Investigation, Validation, Writing – original draft, Writing – review & editing. LY: Supervision, Validation, Visualization, Writing – review & editing. LF: Supervision, Validation, Visualization, Writing – review & editing. CF: Validation, Visualization, Writing – review & editing. HS: Validation, Visualization, Writing – review & editing. XZ: Validation, Visualization, Writing – review & editing. YZ: Investigation, Validation, Visualization, Writing – review & editing. ZS: Supervision, Validation, Visualization, Writing – review & editing.
